# Keith R. Jennings (Ed.): A history of European mass spectrometry

**DOI:** 10.1007/s00216-013-6772-x

**Published:** 2013-02-14

**Authors:** Witold Danikiewicz

**Affiliations:** Laboratory of Mass Spectrometry, Institute of Organic Chemistry, Polish Academy of Sciences, ul. Kasprzaka 44/52, 01-224 Warsaw, Poland


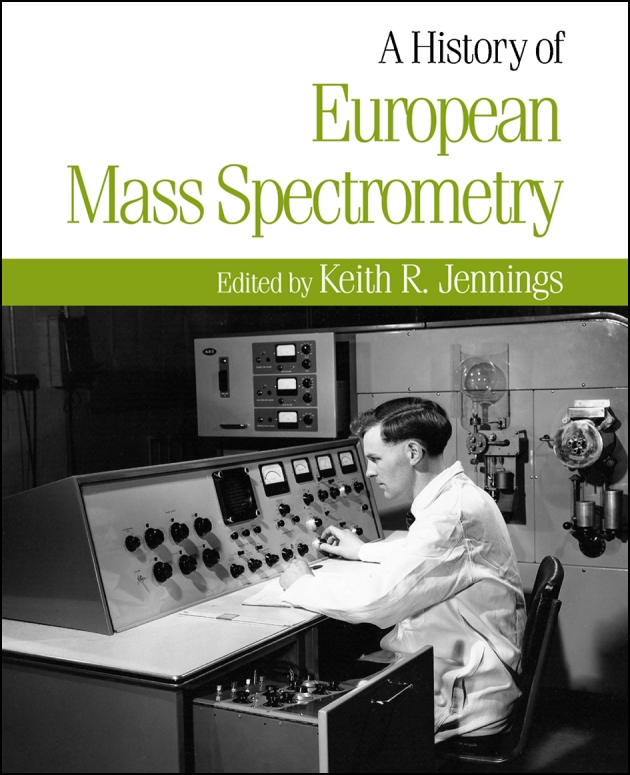


**Bibliography**

A history of European mass spectrometry

Keith R. Jennings (Ed.)

IM Publications LLP

ISBN 978-1-906715-04-5

Hardcover, 285 pages

August 2012, £30.00

**Book’s topic** This book presents the history of mass spectrometry from the point of view of prominent European scientists working in the field and representatives of leading manufacturers of mass spectrometers in Europe. It is by no means a monograph or a textbook but rather a collection of separate essays, written by a number of authors who present their personal thoughts on various aspects of the development of mass spectrometry. This book focuses mainly, though not exclusively, on developments in Europe, which was a cradle of this very important analytical method.

**Contents** This book consists of 11 chapters, written by different authors. It starts with editor Keith R. Jennings’ brief description of the milestones in the development of mass spectrometry, starting from its beginnings in the twentieth century and ending with the most recent developments in the field. The second chapter presents Nico M.M. Nibbering’s views on major European developments in the fundamentals of organic mass spectrometry, largely focusing on the second half of the last century. This author’s own contribution to our present understanding of gas-phase ion-molecule reactions and unimolecular decompositions was great, and there is no doubt he is the right person to describe this rather difficult subject. The next chapter is technical in character, containing Andries Bruins’ description of the long and difficult path which led finally to modern gas and liquid chromatography–mass spectrometry systems. In Chapter 4 Michael Karas describes the development of major desorption ionization methods, from field desorption (FD) through secondary ion mass spectrometry (SIMS), plasma desorption and fast atom bombardment (FAB), ending with matrix-assisted laser desorption ionization (MALDI)—his personal greatest development.

The next two chapters are written by representatives of the manufacturers of mass spectrometers: Bob Bateman and Johen Franzen, from Manchester and Bremen, respectively. They present a fascinating story of the people who, in the middle of the twentieth century, had a vision of mass spectrometry as one of the most useful analytical methods in chemistry. In my opinion this is the most interesting part of the book, providing an eyewitness account of how many obstacles had to be circumvented to reach the level of quality seen in modern mass spectrometers. The next chapter is on a related subject: Michael C. ten Noever de Brauw presents some highlights from the life of Curt Brunnée, one of Bremen’s most important developers of mass spectrometers.

Chapter 8 is by Peter Roepstorff, one of the developers of mass spectrometry applications for the analysis of peptides and proteins. This is another personal story, showing how close personal contacts between researchers in different countries can result in the rapid development of a very difficult—but extremely important—branch of science. The essay by well-known Hungarian mass spectrometrist Károly Vékey describes selected applications of mass spectrometry in analysis of small biomolecules, developed mainly by scientists from Central and Eastern Europe. It is very interesting to realize that despite the serious problems faced by scientists working behind the Iron Curtain, problems which persisted even after the Curtain’s removal in 1989, many of them achieved very important results which are still of great value for today’s researchers.

The author of Chapter 10, Jim Scrivens, points out that the development of mass spectrometry was to a large extent driven by industrial and environmental applications. He shows that modern industry, especially its chemical and pharmaceutical branches, would not exist without analytical methods based on mass spectrometry. Finally, in the last chapter Alison E. Ashcroft presents a short description of scientific societies in Europe which deal with mass spectrometry, as well as mentioning mass spectrometry conferences taking place on that continent.

**Comparison with existing literature** This book is unique—not only because there are no other publications presenting mass spectrometry from the “Europocentric” perspective, but also because there are practically no recent books on the history of mass spectrometry. The only exception is a short book edited by M.A. Grayson: “Measuring Mass: From Positive Rays to Proteins” (Chemical Heritage Press, 2002). Readers interested in the subject can find much information on different aspects of the history of mass spectrometry scattered through chemistry journals and Internet sites, but those sources contain only fragments of the material presented in this book.

**Critical assessment** This book is a collection of essays written by authors from many scientific and industrial environments, so it is rather difficult to assess it critically. There is no doubt that all the authors know perfectly the subjects they are writing about, and this is the strongest point of the book. I believe that any reader’s opinion of this book will depend mainly on the individual preferences and needs of that reader. From my point of view the weakest part of this book is the last chapter, in which some mass spectrometry societies are presented in detail, some are described rather briefly and others, such as the Polish Mass Spectrometry Society, are not mentioned at all.

**Summary** For people who are just starting their adventures in mass spectrometry this book will give a broad overview of this field of science, showing how lucky they are to be working with such advanced and easy to operate instruments. It was not always like this … Veterans in the field will find in this book many names of their good friends—and often scientific competitors—and have the opportunity to find their faces on the vintage photographs. They will also see the instruments, now long dismantled, that were the witnesses of their greatest scientific achievements. In conclusion: every scientist, young or old, who works with mass spectrometry will find something of interest in this book.

